# Recent Advances in Drumstick (*Moringa oleifera*) Leaves Bioactive Compounds: Composition, Health Benefits, Bioaccessibility, and Dietary Applications

**DOI:** 10.3390/antiox11020402

**Published:** 2022-02-16

**Authors:** Piyush Kashyap, Shiv Kumar, Charanjit Singh Riar, Navdeep Jindal, Poonam Baniwal, Raquel P. F. Guiné, Paula M. R. Correia, Rahul Mehra, Harish Kumar

**Affiliations:** 1 Department of Food Engineering & Technology, Sant Longowal Institute of Engineering & Technology, Longowal 148106, India; piyush_pfe1704@sliet.ac.in (P.K.); csriar@sliet.ac.in (C.S.R.); navdeepjindal@sliet.ac.in (N.J.); 2 Department of Food Technology and Nutrition, School of Agriculture Lovely Professional University, Phagwara 144401, India; 3 Food Science & Technology (Hotel Management), Maharishi Markandeshwar (Deemed to Be University), Mullana, Ambala 133207, India; 4 Food Corporation of India, New Delhi 110001, India; pbaniwal@gmail.com; 5 CERNAS Research Centre, Polytechnic Institute of Viseu, 3504-510 Viseu, Portugal; paulacorreia@esav.ipv.pt; 6 Amity Institute of Biotechnology, Amity University Rajasthan, Jaipur 303002, India; rahul.mehra3@s.amity.edu

**Keywords:** *Moringa oleifera*, antioxidants, phytochemicals, bioaccessibility, therapeutic applications

## Abstract

Based on the availability of many nutrients, *Moringa oleifera* tree leaves have been widely employed as nutrients and nutraceuticals in recent years. The leaves contain a small amount of anti-nutritional factors and are abundant in innumerable bioactive compounds. Recently, in several in vivo and in vitro investigations, moringa leaves’ bioactive components and functionality are highlighted. Moringa leaves provide several health advantages, including anti-diabetic, antibacterial, anti-cancer, and anti-inflammatory properties. The high content of phytochemicals, carotenoids, and glucosinolates is responsible for the majority of these activities as reported in the literature. Furthermore, there is growing interest in using moringa as a value-added ingredient in the development of functional foods. Despite substantial study into identifying and measuring these beneficial components from moringa leaves, bioaccessibility and bioavailability studies are lacking. This review emphasizes recent scientific evidence on the dietary and bioactive profiles of moringa leaves, bioavailability, health benefits, and applications in various food products. This study highlights new scientific data on the moringa leaves containing nutrient and bioactive profiles, bioavailability, health benefits, and uses in various food items. Moringa has been extensively used as a health-promoting food additive because of its potent protection against various diseases and the widespread presence of environmental toxins. More research is needed for utilization as well as to study medicinal effects and bioaccesibility of these leaves for development of various drugs and functional foods.

## 1. Introduction

Medicinal plant research and applications are expanding each day due to therapeutic phytochemicals, which can stimulate the progress of novel medicines. Most plant-based phytochemicals, e.g., carotenoids, phenolic acids, flavonoids, tannins, saponins, alkaloids, and glucosinolates, have beneficial effects on well-being and avoidance of malignancy [1]. Phytochemicals are secondary aromatic plant metabolites that prevent disease and are extensively present in plants. They are widely recognized for preventing and reducing chronic diseases risk (e.g., cancer cardiovascular, and neurological) and for beneficial mediation in treating these diseases [2,3].

The drumstick tree (*Moringa oleifera* Lam.) member of the *Moringaceae* family is widely spread from India to Africa and numerous other tropical and arid countries, mainly utilized as food and medicine [4]. Its drought resistance properties, i.e., water-logging of roots, make this plant grow well in drier regions. Moringa plants can grow on different soil types, but well-drained loamy and sandy soil with a pH of 5–9 is best suited for its growth [5]. *Moringa oleifera* is viewed as a most valuable plant because all parts can be utilized for food, medication, and other industrial and household purposes [6,7]. The leaves, in particular, may be consumed as a salad, roasted, or stored as dried powder for a long period without losing nutritious content. Besides utilizing its leaves for food and feed, because of inborn phytochemicals like phenolic acids, flavonoids, carotenoids, and glucosinolates, they also have potential applications as functional foods nutraceuticals [8,9]. Crypto-chlorogenic acid, isoquercetin, and astragalin are the significant phytochemicals present in moringa leaves which are attributed to the antioxidant, anti-hypertension and anti-inflammation activities [10,11]. The medicinal functions and biological activity of these plants extract have been predominantly upheld by various in vitro assays based upon the bioactive components and their antioxidant activity [8,9,10,11,12,13]. Its high phenolic content is primarily responsible for its antioxidant effects. Different pharmaceutical products from this plant have been manufactured and sold in both the Indian and worldwide markets due to these medicinal advantages [14,15].

*Moringa oleifera* is also called “Miracle Tree” or “Tree of life”, owing to its excellent health, nutritional and environmental effects. Traditionally, moringa leaves are used as medicine in India to cure conjunctivitis and also to remove intestinal worms from the abdomen [9]. The fresh moringa leaves also improve the milk production of pregnant and lactating mothers and are used to treat anemia [16]. Diabetic patients can also use moringa leaves juice to control blood pressure and blood glucose levels. Moringa processing may sometimes alter the bioaccessibility of moringa nutrients and polyphenols. Therefore, new approaches are needed to increase polyphenol retention when moringa leaves are processed and stored.

Recently, the usage of herbal medicine has been increased exponentially. Developing countries depend basically on therapeutic plants for their wellbeing needs. Consequently, moringa leaves are a suitable option in developing nations looking for quality health services that offer inexpensive and easily accessible treatment in places not accessible to Western medicine. The proper dietary consumption knowledge by medical science experts helps in slowing the growth of many diseases. Since no aggregated data on moringa leaves are available revealing the vital bioactive components, bioaccessibility and health benefits, this review intends to fill a void in the scientific literature. Thus, the study focuses primarily on current knowledge on *Moringa oleifera* leaves’ nutritional content and composition of bioactive compounds, their bio-accessibility, and health-promoting effects. It also allows researchers to broaden their research and explore moringa leaves as functional foods in various food products.

## 2. Nutritive Composition of *Moringa oleifera* Leaves

*Moringa oleifera* is considered as miracle tree because it is extensively used as a nutritive herb with high nutritional content and a food supplement to overcome child malnutrition [9]. A complete nutritional profile of *Moringa oleifera* leaves is shown in Table 1. The crude protein content of leaves varied from 10.74% to 30.29%, carbohydrate from 13.41 to 63.11%, fat from 6.50 to 20%, crude fiber 7.09 to 35%, and mineral matter from 7.64 to 10.71% on the dry weight basis [16,17]. Moringa leaves have an exceptionally high amount of protein as compared to other leaves, being consumed as food. *Moringa oleifera* also contains essential amino acids and a high amount of provitamin A [18]. The nutritional content of moringa varied based on the climacteric condition, and among cultivars, e.g., *Moringa oleifera* leaves grown in different areas of Thailand contain different nutritional profiles [19]. Its protein content ranges from 19 to 29% and fiber content from 16 to 24%. Similar findings have been reported by Teixeira et al. [20] in Brazil and Moyo et al. [17] in South Africa, with samples showing a protein content of leaves of approximately 28% and 30%, respectively. An amount of 100 g of fresh moringa leaves contains 17.5% of the daily required level of protein. Among the fatty acid profile of moringa leaves, it contains the maximum amount of unsaturated fatty acid, with α-Linolenic acid being the largest among them [17]. Recently, a new polysaccharide was isolated from moringa leaves named MOP-2 through hot water extraction, and various chromatographic techniques have been used for its purification. This MOP-2 may be used as an immunoregulatory agent in various functional foods [21]. Moringa leaves are also plentiful source of polyunsaturated fatty acids such as omega-3 and omega-6, making them essential in various cardiovascular functions and vitalizing the body. It also contains less saturated fatty acids and a high amount of monounsaturated fatty acids [22].

The unsaturated and saturated fatty acids in leaves were 57% and 43%, respectively, with α-linolenic acid the most prominent unsaturated fatty acid [17]. Moreover, it has also been reported that leaves contain 16–19 amino acids, out of which 10 are essential amino acids, that is lysine, leucine, isoleucine, histidine, phenylalanine, methionine, tryptophan, threonine, tyrosine and valine [6]. The calorific value of moringa leaves is also low; thus, it can be used by obese persons.

Moringa is considered to be a good source of nutrients that are necessary for growth and development. Moringa leaves, which contain four times more calcium and two times more digestible protein than milk, can be used as calcium and protein supplements. The moringa leaves are also rich in minerals such as potassium, zinc, magnesium, iron and copper [23]. Iron tablets can be replaced with moringa powder to treat the disease called anemia. The amount of iron in beef and leaf powder is 2 mg and 28 mg, respectively, more than spinach [16]. Fat-soluble vitamins such as vitamin-A (pre-cursor of beta-carotene), D and E; water-soluble vitamin-B complexes such as folic acid, pyridoxine and nicotinic acid; and vitamin-C, are also present in *M. oleifera* [24]. Vitamins A and C present in fresh leaves are 7564 IU and 145 µg, respectively, which is 252% and 235% of the daily required vitamin A and C levels. When malnourished children were administered 10 g of dried moringa leaf powder daily, a significant increase in weight gain was reported and promoted rapid recovery compared to control in 6 months. The *M. oleifera* leaves have adequate sources of phytochemicals such as phenolic acids, flavonoids, tannins, saponins, alkaloids, etc., and their derivatives are known for their anti-cancerous properties [25].

Saini et al. [26] reported that an appreciable number of carotenoids (trans-lutein (approximately 30%), trans-b-carotene (approximately 18%), trans-zeaxanthin (approximately 6%) are present in fresh leaves. Along with carotenoids, good amounts of tocopherol (36.9 mg/100 g) and ascorbic acid (271 mg/100 g) are also present in leaves. With this nutritional composition *Moringa oleifera* also contains a trace amount of antinutrients such as phytates, saponins, tannins, and oxalates [27]. These are not toxic or pernicious. When taken in high amounts, they may interfere with the assimilation and ingestion of different supplements, such as zinc, iron, calcium, and magnesium. Its seeds and leaves contain less phytate and saponins than most legumes such as soybean. For that reason, leaves are found to be nutritionally safer and healthier for consumption [28,29].

## 3. Bioactive Profile of *Moringa oleifera* Leaves

**Figure 1:**
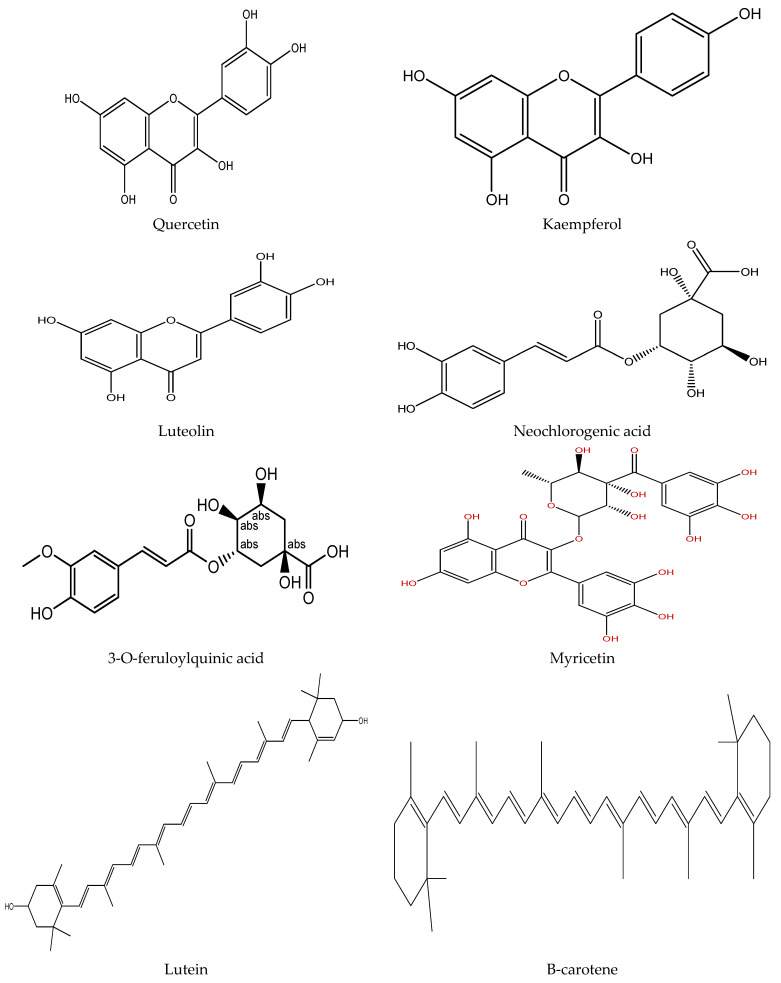

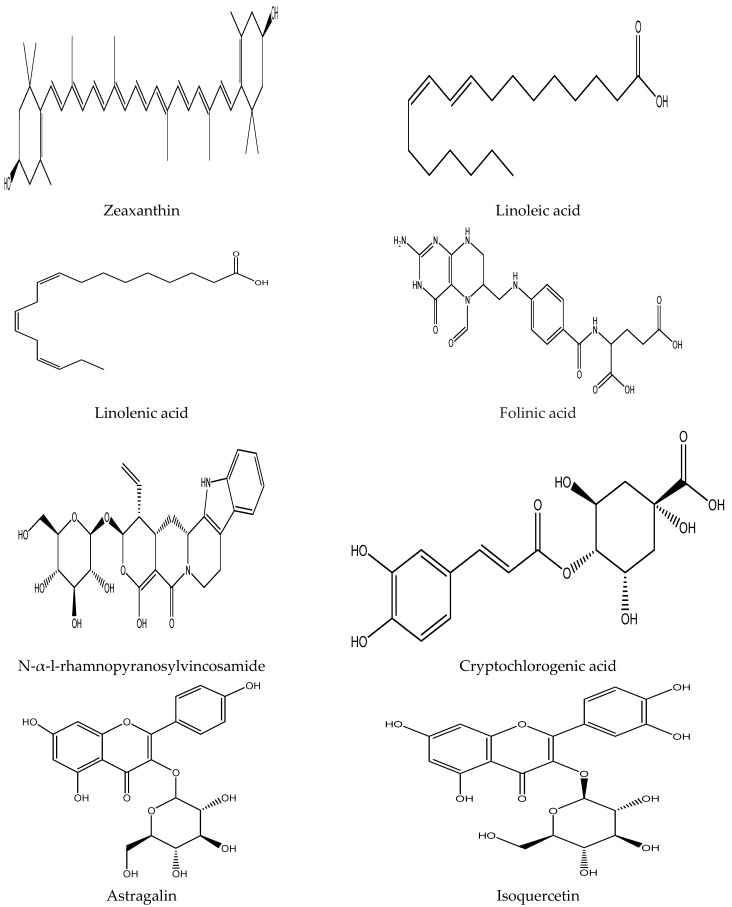
Chemical structure of some bioactive compounds presents in *Moringa oleifera* leaves.

Plants contain various chemical compounds like phenolic acids, isothiocyanates, tannins, flavonoids, and saponins, which are physiologically active and utilized in food materials. These compounds are therapeutically active or inactive. They are synthesized by plants to combat environmental and physiological stresses such as ultraviolet radiation and microbial attack [30,31]. The *Moringa oleifera* is an important plant with several bioactive compounds present in its leaves, such as flavonoids, saponins, tannins, catechol tannins, anthraquinones, alkaloids (Figure 1). These properties make moringa leaves beneficial for nutritional and therapeutic applications, as well as a water purifying agent (Table 2).

### 3.1. Phenolic Compounds

Phenolic compounds are plant secondary metabolites mostly present as derivatives of hydroxycinnamic acid (free-phenolics) and hydroxybenzoic acid (bound-phenolics). These compounds have one or more hydroxy groups that are directly connected to the aromatic ring and can be found in plant material as esters or glycosides [41]. These hydroxyl groups are responsible for the high scavenging activity of phenolic compounds [42]. Moringa plant was found to have several phenolic compounds, and their bioactivity was confirmed by both in vitro and in vivo analysis. The major phenolic compounds found in leaves are lignans (i.e., medioresinol, isolariciresinol, secoisolariciresinol and epipinoresinol glycosides), 26 flavonoids (i.e., quercetin, kaempferol, apigenin, luteolin and myricetin), and 11 phenolic acids and their derivatives (i.e., caffeoylquinic, feruloylquinic, and coumaroylquinic acids and their isomers) [36,42]. The total phenolic content in methanolic extract of moringa leaves varied from 71.08 ± 12.05 to 76.63 ± 10.63 mg GAE/g [36], and the concentration was 22% more than that from young leaves of *M. Peregrina* [43]. This makes *Moringa oleifera* leaves a better source of these phytochemicals. Moringa plants’ phytochemical composition depends on germplasm, maturity stage, and agroclimatic conditions [9,37]. Along with these, phytochemical compositions also depend on the storage condition as well as storage time. Vongsak et al. [11] conducted a study at two different temperatures (25 ± 2 °C and 40 ± 2 °C) and relative humidities (60 ± 5% and 75 ± 5% RH) for six months, and found that at 25 ± 2 °C and 60 ± 5% RH, there was a slight decrease in bioactive content (13–27%) and DPPH (30%) radical scavenging activity whereas, at 40 ± 2 °C and 75 ± 5% RH, bioactive content significantly decreases from 38 to 53%, whereas antioxidant capacity decreases by 50%. All the samples were stored in aluminum foil bags.

The major phenolic chemicals identified in moringa leaves are flavonoids [36]. Some of the major flavonoids found in leaves are quercetin, kaempferol, apigenin, luteolin, and myricetin glycosides. *Moringa oleifera* leaves predominantly contain quercetin (43.75%) and equal percentages (18.75%) of other flavonoids [34]. The higher concentrations of quercetin (1362.6 mg/Kg) and kaempferol (1933.7 mg/kg) were found in moringa leaves as compared to spinach quercetin (17.9 mg/Kg) and kaempferol (215.3 mg/Kg) [44]. The concentration of flavonoids varied with the environmental conditions. The UHPLC-ESI-q-TOF-MS study revealed that 17 different flavonoids were found in the leaves of *Moringa oleifera* harvested from South Africa and Namibia region with quercetin (35%), kaempferol (35%), isorhamnetin (24%) and apigenin (6%) derivatives [45], whereas 12 flavonoids were detected in sub-Saharan African region leaves through HPLC-UV-MS [46].

Moringa leaves contain 77 to 187 µg per gram DM of phenolic acids with hydroxybenzoic acids and hydroxycinnamic acid derivatives [37]. The caffeoylquinic acid (45.45%), coumaroylquinic acid (36.37%) isomers, [8,36] and hydroxybenzoic acids (gallic acid and p-hydroxybenzoic acid) [36,37,43] are the major phenolic acids present in *Moringa oleifer* leaves. In a recent study, 63 phenolic acids (mainly hydroxycinnamics) were found in moringa leaves, from which gallic acid and chlorogenic acid were the most abundant phenolic acids [34]. The presence of cis and trans-3-acyl, 4-acyl, 5-acyl, caffeoylquinic, p-coumaroylquinic and feruloylquinic acids were reported for the first time in *Moringa ovalifolia* [45].

### 3.2. Carotenoids

Carotenoids are lipophilic molecules that are naturally occurring pigments synthesized by photosynthetic plants, preventing excess energy damage to photosynthetic apparatus [47]. These pigments function as antioxidant chemicals, giving a variety of health advantages such as protection from cellular damage, ageing, and other chronic illnesses. These can also be used as popular dietary supplements such as food colorants. *Moringa oleifera* leaves have abundant carotenoids with a total amount varying from 44.30 to 80.48 mg/100 g on a fresh weight basis among eight different cultivars. The six different carotenoids that are mainly found in leaves are luteoxanthin, 15-Z-β-carotene, 13-Z-lutein, β-carotene, all-E-β-carotene, all-E-lutein, and all-E-zeaxanthin. All E-β-carotene and luteoxanthin have maximum and minimum purity in a purified carotenoid extract with 89 and 94%, respectively [31]. Phullakhandam and Failla [48] reported that lutein and β-carotene concentrations in fresh leaves are 418 and 272 mg/kg of dry weight, respectively, whereas in dried powdered leaves, it is 472 and 166 mg/kg, respectively. Environmental factors, post-harvest conditions, plant developmental stage, and cooking treatments adversely affect the quantity of carotenoids in the leaves. At the early developmental stage, plants have the highest carotenoids level, which decreases as plant growth progresses, and post-harvest storage at 0 °C also protects carotenoid levels [49]. Cooking at high temperatures also decreases carotenoid levels [50]. Carotenoids as potential antioxidants are attracting interest in terms of decreasing the incidence of certain types of cancer.

### 3.3. Alkaloids, Glucosinolates and Iso-Thiocyanates

Among different plant-derived secondary metabolites, alkaloids are extensively distrusted containing basic nitrogen atoms. N,α-L-rhamnopyranosylvincosamide, phenylacetonitrilepyrrolemarumine, 40-hydroxyphenylethanamide-α-L-rhamnopyranoside and its glucopyranosyl derivative are the major alkaloids present in *Moringa oleifera* leaves [14,51]. These alkaloids and their derivatives are extensively used for the treatment of various medical disorders. Glucosinolates are another type of secondary metabolites found in leaves and seeds, in which 4-O-(a-L-rhamnopyranosyloxy)-benzylglucosinolate (glucomoringin) is the major one [8]. A natural plant enzyme, myrosinase, generates isothiocyanates, nitriles and thiocarbamates renowned for their powerful hypotensive and spasmolytic effects through enzymatic catabolism [9].

### 3.4. Other Compounds

Other major bioactive ingredients in *Moringa oleifera* leaves are folates, tannins, saponins and fatty acids. Folate, a vital water-soluble vitamin, is a key component of many cell metabolisms [52]. 5-Formyl-5,6,7,8-tetrahydrofolic acid, 5,6,7,8-tetrahydrofolic acid, 5-Methyl-5,6,7,8-tetrahydrofolic acid, and 10-Formylfolic acid are the primary forms of folates present in *Moringa oleifera*. According to RDA, the bioavailability of natural folates is only 50%, whereas moringa folates’ bioavailability was found to be 81.9% studied in a rat model [31]. Thus, *Moringa oleifera* and derived foods are important sources of folates due to their higher bioavailability.

Moringa leaves are also rich sources of ω-3 and ω-6 polyunsaturated fatty acids, whereas α-linolenic acid (49–59%) and linoleic acid (6–13%) are the major polyunsaturated fatty acids. Palmitic acid is the primary fatty acid among saturated fatty acids, with the amount varying from 16 to 18% of total fatty acids present in leaves. The *M. oleifera* leaves have higher polyunsaturated fatty acids and lower monounsaturated fatty acids than its pods [28]. Tannins are the water-soluble polyphenolic astringent biomolecules precipitating proteins, alkaloids and other organic molecules with a concentration varying from 13.2 to 20.6 g tannins/kg in the dry leaves [20]. Saponins are the other organic compounds in *Moringa oleifera* leaves made up of isoprenoid-derived aglycone, covalently linked to sugar moieties [53]. Its freeze-dried leaves content varied from 64 to 81 g/kg dry weight [29]. Both tannins and saponins are reported to possess various therapeutic properties [54].

## 4. Health Benefits of *Moringa oleifera* Leaves

**Figure 2:**
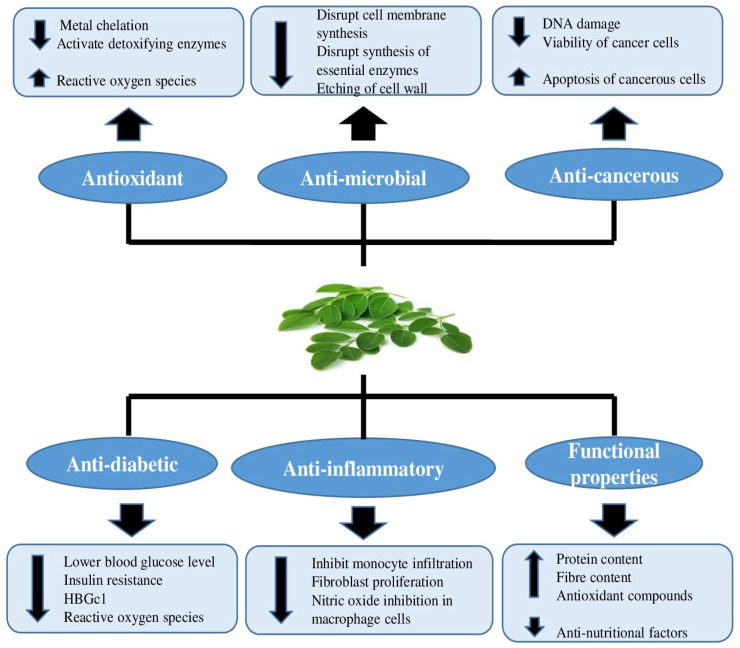
Health benefits of *Moringa oleifera* leaves based on their inherent properties.

Moringa leaves offer multiple health advantages, including antioxidant activity, anti-microbial activity, anti-cancerous activity, anti-inflammatory action, and many more, as shown in (Figure 2) [10,11].

### 4.1. Antioxidant Properties

Reactive oxygen species oxidize biological molecules by overcoming the cell’s antioxidant defense mechanism and thus inducing damage to cell membranes, proteins, carbohydrates, and DNA. Hypertension, diabetes, heart failure, and several pathological situations are the cause of this oxidative stress [55]. Natural antioxidants are always the first choice of consumers and are better than synthetic antioxidants [56]. Quercetin, kaempferol [57], ascorbic acid, β-carotene [58], isothiocyanates, polyphenols, and rutin [59] are potent antioxidants found in the leaves of *Moringa oleifera*. Leaves’ extracts in various organic solvents such as methanol, acetone, dichloromethane, water, diethyl ether, chloroform, and ethyl acetate have been found to have antioxidant properties [10,60,61]. The ethyl acetate extract of *Moringa oleifera* has greater scavenging activity for the superoxide anion radical (O_2_.^−^) to prevent interaction of active free radicals with biological macromolecules and hence reduces the damage to tissues which occurred [10]. The higher antioxidant activity of leaves has a linear relationship with phenolic compounds [61], which helps develop products that enhance food products’ oxidative stability. *Moringa oleifera* methanolic leaves’ extract showed decent antioxidant activity (IC_50_ 49.86 µg/mL) compared with ascorbic acid (IC_50_ 56.44 µg/mL) due to the presence of higher polyphenolic content [62]. It has also been reported that leaves’ antioxidant profile concurs with the cryoprotective nature of plants [63], and its parts are used as a natural preservative for fat [64]. *Moringa oleifera* tea with antioxidant potential (81% inhibition of DPPH radicals compared with vitamin-C (0.1 mg/mL) with 76.5% inhibition) might be helpful in preventing stress-related chronic disorders [65]. Recently, Khalofah et al. [66] studied that moringa leaf extract significantly reduced the negative effect of cadmium stress on *Lepidium sativum*. A moringa extract dosage of 100 mg/kg body weight effectively increases the antioxidant levels in aluminum phosphide-intoxicated rats and reduces malondialdehyde (MDA). Thus, it can be used as adjuvant therapy against aluminum phosphide (AlP)-induced cardiotoxicity [67].

Alavrez-Roman et al. [68] used the hydroalcoholic fraction of moringa leaves for the preparation of topical formulations (nanoparticles and gel) and determined their phytochemical profile moisturizing and antioxidant potential. Both formulations showed good viscosity, pH and particle size, confirming their suitability as a formulation. Seven different compounds were identified, including flavonoids and phenolic acids. Moreover, higher antioxidant activity and good skin biophysical evaluation results (higher stratum corneum water content and lesser trans-epidermal water loss) showed that this formulation could be used as a new skin drug delivery system. In another study, moringa leaves were used as a replacement for alfalfa hay to increase milk and serum quality of goats [69]. Their analysis includes three diets with alfalfa alone, 25% *Moringa oliefera* leaves and 25% *Moringa peregrine* in the diet of goat’s fodder. Ten goats in each experiment were used, and each experiment includes an adaption of two weeks and a collection of six weeks data. Goats fed with both types of moringa leaves exhibited greater fat content, free from nitrogen extract and total phenols, than alfalfa alone diet. Moreover, moringa feed goats have improved the oxidative status of serum and milk by enhancing total antioxidant activity, vitamin C, catalase activity, and decreased thio-barbituric acid reactive substance (TBARS) concentration [69].

Saleem et al. [70] studied the in vitro antioxidant activity at different concentrations (0.1563–5 mg/mL) of various extracts of moringa leaves. They found that all the extracts showed radical scavenging activity at a low concentration of 0.1563 mg/mL and methanolic extract showed maximum DPPH activity at all different concentrations. Similarly, the methanolic extract showed the highest H_2_O_2_ scavenging activity (70.56 ± 0.43%) and reducing power (925.48 ± 0.45%) at 1 mg/mL concentration. This high antioxidant potential of methanolic extract is due to higher total phenolic content (TPC) and total flavonoid content (TFC) than other extracts.

The impact of methanol extract from moringa leaves in the heart of diabetic rats caused by oxidative stress produced by streptozotocin has been investigated by Aju et al. [71]. The rats were fed orally with moringa leaves at 300 mg/Kg body weight concentration for 60 days. They were categorized into six groups, i.e., normal control rats (group 1), normal rats treated with moringa leaves (group 2), high energy diet control rats (group 3), diabetic control rats (group 4), diabetic rats treated with moringa leaves (group 5), and diabetic rats treated with metformin and atorvastatin (group 6). The authors concluded significant decrease in antioxidant enzymes, such as catalase (CAT), glutathione (GSH), glutathione peroxidase (GPx) and superoxide dismutase (SOD), activity in rats from groups 3 and 4, whereas antioxidant enzymes activity in the heart of rats were increased in groups 2, 5 and 6 rats. Various antioxidant compounds such as hexadonic acid, phytol, DL-alpha-tocopherol and other compounds in moringa leaves are responsible for their antioxidant potential.

Recent studies showed that researchers used both in vitro cultural models and in vivo animal models to display the potent health benefits of *Moringa oleifera* leaves (Table 3).

### 4.2. Antimicrobial Activity

As resistance strain incidences of pathogens increase, resulting in higher death rates worldwide, new and improved antimicrobial drugs must be developed [85,86]. In this regard, medicinal plants are coming into the limelight, having a superior approach to health and being devoid of synthetic pharmaceutical side effects. The leaf extracts of *Moringa oleifera* have been tested against *Escherichia coli*, *Staphylococcus aureus*, *Bacillus subtilus*, *Salmonella typhi*, *Pseudomonas aeruginosa*, *Proteus vulgaris*, *Helicobacter pylori*, *Klebsiella pneumonia*, Micrococcus Kristina for antimicrobial activity [72,87,88,89]. *Moringa oleifera* leaves have strong antimicrobial activity except against *P. aeruginosa*, which is resistant to aqueous leaf extract. An array of phytochemicals is responsible for the antimicrobial activity of leaves [72].

Suarez et al. [90] and Bukar et al. [91] identified a short peptide 4 (ά-L-rhamnosyloxy) benzyl-isothiocyanate in leaves and argued that it might inhibit the growth of microorganisms through disruption of synthesis of the cell membrane or important enzymes. Methyl N-4-(α-Lrhamnopyranosyloxy) benzyl carbamate, 4-(α-D-glucopyranosyl-1–4 α-L-rhamnopyranosyloxy)-benzyl thiocarboxamide [92], 4-(α-L-rhamnopyranosyloxy) benzyl glucosinolates [93], also responsible for the antimicrobial activity of leaves of *Moringa oleifera*. Antifungal activity of steam distillate moringa leaves was tested against *A. niger*, *A. oryzae*, *A. nidulans* and *A. terreus* and it was observed that *A. niger* shows maximum inhibition, due to the presence of a large number of phytochemicals in the moringa distillate [72]. Ishnava et al. [94] reported that methanolic leaf extract showed the highest antifungal activity (25 mm) against *Tricodermaharzianum.* Bioactive compounds present in leaves extract might serve as a natural antimicrobial agent. It has also been reported that a chemical compound, pterygospermin, is present in moringa leaves, which is dissociated into two molecules of antimicrobial benzyl isothiocyanate [95].

Rocchetti et al. [35] also found that methanolic moringa extract showed a maximum zone of inhibition against *Listeria innocua* (10.21 ± 0.08 mm), *Salmonella enteritidis* (5.67 ± 0.47 mm), *Salmonella typhimurium* (5.33 ± 0.47 mm) and in the case of *Bacillus cereus* (15 ± 0.00 mm) a 50–50 v/v methanol–water mixture showed maximum inhibition. In contrast, acetone and ethanol extracts of leaves showed good antimicrobial activity against *Bacillus subtilis* (MIC:0.78 mg/mL, 0.78 mg/mL)*, E. coli* (MIC:0.78 mg/mL, 0.78 mg/mL) and *Staphylococcus aureus* (MIC:0.78 mg/mL, 0.39 mg/mL) and high MIC value was reported against *Bacillus subtilis* (MIC:1.56 mg/mL) for the water extract of moringa leaves [22].

Prabhakaran et al. [39] studied the antimicrobial activity of various solvents (methanol, water, acetone, ethanol, and ethyl acetate) from different plant parts (leaves, flowers, roots, seeds and bark) of *Moringa oleifera*. The disc diffusion method was used to study the antibacterial effect against *P. aeruginosa* and *E. carotovara*. They found that ethanol, methanol, and ethyl acetate extract of leaves showed inhibitory activity against both bacteria. Ethanolic leaves’ extract showed good minimum inhibitory concentration (MIC) (79 ± 0.3%). The high total phenolic content and the presence of various phenolics are responsible for significant antibacterial activity. These polyphenols interact with the protein and enzymes of the cell membrane and destroy the cell membranes structures, thus inhibiting cell functions and leading to the death of microbes [96].

Dose-dependent antibacterial activity of 70% moringa leaves ethanol extract was studied against *Staphylococcus epidermis* and a significant difference was observed with an increase in the concentration of extract [97]. Another study used different concentrations of aqueous and ethanol extract of leaves (75, 50 and 25 mg/mL) against *E. coli*, *Salmonella typhimurium*, *Shigella* spp., *Staphylococcus aureus* and *Enterococcus faecalis* isolated from patient’s stool attending Yobe State Specialty Hospital Damaturu and found that ethanol extract showed higher antimicrobial activity and also the highest zone of inhibition for all organisms observed at 100 mg/mL [98]. The impact of aqueous, methanol, and ethanol extract from the *Moringa oleifera* and *Matricaria recutita* leaves against 40 susceptible antibiotic strains and bacterial resistance strains have been analyzed by Atef et al. [99]. They found that aqueous and methanol extracts of both plants exhibited good activity for all strains, but more activity was observed in the case of moringa leaves extract. However, Bancessi et al. [100] reported that distilled water and 95% ethanol extract of leaves showed higher antimicrobial activity against contaminated drinking water pathogens than other parts of the moringa plant.

In another study, ethanolic and water extracts of moringa leaves were used to study the antimicrobial activity against different pyogenic bacteria isolated from dromedary camel abscesses. They found that both extracts showed good antimicrobial activity against all the bacteria. However, the ethanolic extract was found to be better with a high zone of inhibition, i.e., 25.65 ± 0.04, 30.5 ± 0.28, 26.75 ± 0.04, 27.75 ± 0.04, 28.5 ± 0.3, 19.5 ± 0.05, 24.75 ± 0.12, 22.25 ± 0.05 mm against *Corynebacterium pseudotuberculosis*, *Corynebacterium ulcerans*, *Staphylococcus aureus*, *E. coli*, *Klebsiella pneumoniae*, *Citrobacter* spp. *Proteus vulgaris*, *Pseudomonas aeruginosa*, respectively [101]. Moringa leaves aqueous extract also reported good antifungal activity against *Aspergillus niger* (15.2 ± 0.52 mm), *Aspergillus flavous* (12.4 ± 0.55 mm), *Penicilliumitalicum* (10.5 ± 0.26 mm), *Fusarium oxysporum* (9.4 ± 0.71 mm), *Rhizopus stolonifera* (13.2 ± 0.58 mm), *Anternaria* sp. (6.6 ± 0.47 mm), *candida albicans* (12 ± 0.44 mm) *and Candida parapsilosis* (18 ± 0.54 mm) [4]. These studies suggest that *oleifera* leaves extract inhibits microbial growth by either blocking or bypassing the pathogens resistant mechanism, and thus helps eradicate microbial growth [102].

### 4.3. Anti-Cancerous Properties

Cancer is considered a leading cause of fatalities worldwide, with one out of six deaths occurring due to cancer [103]. Traditional, widely used medicines and treatments for cancer include radiation, chemotherapy, and surgery. All these treatments are costly and have multiple side effects too. Hence medicinal plants are being focused on by the scientific community because of their highly effective phytochemicals. *Moringa oleifera* is a powerful anti-cancer agent as its usage within a limited scale is safe, natural, and reliable [104]. It has been reported that quercetin, kaempferol [105], (4-[(4′-O-acetyl-α-L-rhamnosyloxy) benzyl] isothiocyanate, O-ethyl-4-(α-L-rhamnosyloxy) benzyl carbamate, 4-(L rhamnosyloxy) benzyl isothiocyanateniaziminin and niazimicinfrom the leaves have anti-cancerous activities [9]. They prevent the proliferation of cancer cells and hence can be used as anti-neoproliferative agents. Reactive oxygen species (ROS) produced by moringa extract are target-specific, making them potent anti-proliferative agents that target the cancer cells. *Moringa oleifera* leaves were found to have anticancerous activity against HeLa cells by activating the apoptotic pathway [106]. Berkovich et al. [31] studied pancreatic cancer cell growth inhibition due to moringa leaves. Al-Asmari et al. [107] studied the effect of moringa leaves, bark, and seed extract against MDA-MB-231 and HCT-8 cancer cell lines and it was found that leaf and bark have remarkable anti-cancerous activity. A seven-fold increase in apoptotic cells of MDA-MB-231 breast cell lines and a several-fold increase in apoptotic HCT-8 colorectal cancer cell lines were observed. Another study found that moringa leaves extract helps induce apoptosis by upregulating BAX and downregulating BCL-2 expression, enhancing caspase-3-activity [83]. D-allose and hexadonic acid (palmitic acid) present in leaves are responsible for inhibiting cancer cell growth [108]. At the G1 phase (G1-cell cycle arrest), D-allose induces specific thioredoxin interacting protein (TXNIP) and stabilize p27kip1 protein, which inhibits the cancer cells growth without affecting normal cells [108].

The anticancer activity of moringa leaves aqueous extracts was investigated against human hepatocellular carcinoma HepG2 cells. A significant reduction (44–52%) was seen in HepG2 cell growth when the leaf extracts were orally administered, making them potent anticancer agents [109]. Another study suggested that the bioactive and dietary compounds present in moringa leaves may have a chemoprotective effect and thus significantly suppressed the AOM/DSS-induced colorectal carcinogenesis [32]. A methanolic extract of moringa leaves also showed an anti-cancerous effect in the human prostate cancer DU145 cell line. Inhibition of cell survival and nuclear alteration was dose-dependent, and cell apoptosis was caused by upregulation and downregulation of Bax and Bcl-2 gene expression, respectively, concurrently. Moreover, moringa extract also downregulated the Notch-1 and Hes-1 expression to suppress the abnormal notch signaling pathway [110].

Madi et al. [111] performed various assays to study the mechanism of action of moringa leaves against A549 lung cancer cells. Reactive oxygen species (ROS) levels were significantly increased with increased leaf concentration in the p-nitro-blue-tetrazolium assay, thus provoking apoptosis of the cancer cells. The ATP bioluminescence and ApoGSH colorimetric assay showed that ATP and Glutathione levels significantly decreased with increased leaf extract concentration. These results suggest that the mitochondrial pathway was affected by leaf extract, causing cell death. Western blotting confirmed increased expression of apoptotic markers indicating higher cell apoptosis. Then, FLICA assay was conducted to evaluate cell apoptosis, and after 24 h treatment, most of the cells were fluoresced, suggesting active caspase and cell apoptosis activated. Thus, it can be concluded that moringa leaf extract induces mitochondrial membrane depolarization, which leads to a decrease in ATP level. This higher ATP level increases the amount of ROS and decreases GSH, which causes cell death. Sadek et al. [112] studied the chemo-prophylactic effect of moringa leaf extract against diethyl nitrosamine (DEN)-induced hepatocellular carcinoma in Wistar male rats, which were fed with leaf extract (500 mg/kg) for one week and then with leaf extract and Den (10 mg/kg) for 16 weeks. The results allowed inference that administration of moringa leaves enhanced hepatocellular appearance, the DEN-induced elevations in serum biochemical records were significantly decreased, and the 8-OHdG level was decreased by 29%. Bax and caspase-3 expression was enhanced, but Bcl-2, Bcl-xl, and β-arrestin-2 expression were downregulated. This might be due to increased ROS production or moringa leaves with critical defensive impacts against DEN-induced hepatocarcinogenesis, leading to apoptosis actuation.

In another study, the anticancerous activity of moringa leaves was invested against AOM/DSS induced colorectal cancer in a male mice model. Four different groups of mice model were prepared with negative control (no AOM/DSS) (Group 1), positive control (10 mg/kg body weight AOM and 3 cycles of 2% DSS) (Group 2), AOM/DSS and 2.5% moringa leaf powder (Group 3) and AOM/DSS and 5% moringa leaf powder (Group 4). The activity of harmful fecal enzymes tryptophanase, β-glucosidase, β-glucoronidase, and urease was significantly decreased by 103%, 40%, 43%, and 266%, respectively, with 5% moringa leaf powder dose. Histopathology study showed that supplementation of 2.5% and 5% moringa leaf powder to mice induced chemoprotective effect via crypt deformation and reduction in the formation of adenoma, and incidence of tumors was reduced by 50% with a higher moringa dose [32].

Barhoi et al. [113] investigated the anti-carcinogenic potential of moringa leaves by both in vitro and in vivo assays. Ehrlich ascites carcinoma (EAC) and human laryngeal carcinoma (Hep-2) cells were used for in vitro studies, whereas Balb/c mice were used for in vivo studies. Mice received Mitimycin C (MMC) at a dose of 2 mg/kg body weight and aqueous moringa extract at 200 and 400 mg/kg body weight. Reduction in the tumor was dose-dependent, with 78.69% tumor volume reduced at a dose of 400 mg/kg body weight, whereas 36.97% reduction was verified at a dose of 200 mg/kg body weight after 50 days. Similarly, tumor weight was also significantly decreased at doses of 200 mg/kg (12.45 ± 1.20 g) and 400 mg/kg (8.43 ± 0.49 g) of moringa as well as 2 mg/kg MMC (14.42 ± 1.09 g) as compared to the control (27.91 ± 1.50 g). In vitro analysis also showed dose and time-dependent toxic effects on both the cell lines.

Methyl isothiocyanate, an important bioactive compound present in moringa leaves, was investigated against TPA-mediated carcinoma in JB6 cells of mouse epidermis. DNA methyl seq and RNA seq technology were used to identify differentially mediated regions (DMRs) and differentially expressed genes (DERs). Results showed that methyl isothiocyanate reversed the expression of several DMRs and DERs. The study also revealed that several inflammatory and Nfr-2 mediated antioxidative and tumor-suppressive pathways are restored by methyl isothiocyanate that was upregulated and downregulated by TPA [114]. These studies suggest that *Moringa oleifera* leaves contain various phytochemical constituents, potent anticancerous agents and could be used to design functional foods.

### 4.4. Antidiabetic Properties

Diabetes mellitus (DM), a chronic disease due to insulin and its action deficiency or both, leads to delayed hyperglycemia, ultimately affecting metabolic processes inside the human body [115,116]. If untreated, it will severely cause tissue and vascular damage, prompting serious complications and retinopathy [117], neuropathy [118], nephropathy [119], cardiovascular complications, and ulceration [120]. The World Health Organization (WHO) stated that approximately 150 million people worldwide suffer from Diabetes Mellitus. The number is expected to reach up to 300 million by 2025 [121]. Type-1 and Type-2 are two types of diabetes. An absolute deficiency of insulin secretion characterizes Type-1 diabetes, so they need insulin substitutes [122]. Type-2 (non-insulin dependent diabetes mellitus (NIDDM)) diabetes is most common and occurs due to abnormal insulin secretion and its resistance [123].

In another study, 100 Type II diabetic patients were provided with a tablet formulated with 98.34% dehydrated *Moringa oleifera* leaf powder in a private clinic, and it was found that after 90 days of trials, postprandial blood glucose and glycosylated hemoglobin was reduced up to 28.57% and 7.4%, respectively, as compared to the initial value [124]. Similarly, another formulation of leaf powder supplemented with 5% salt, 7% red chilli powder, and 7% coriander powder and slightly fried without oil was made and supplied to type-II diabetes mellitus obese patients. They were given 50 g pouches and advised to use them for 40 days regularly with food. In diabetic individuals, the prepared leaf powder dramatically reduced serum blood glucose [125]. Blood glucose levels are reduced by phenolics and by other antioxidant substances in the blood.

Several studies showed that moringa leaves can cure both types of diabetes. The aqueous extract from *Moringa olieifera* leaves might be used to treat Type I and insulin resistance Type II diabetes in a research investigation on streptotocin-induced rats [126]. Due to ATP dephosphorylation caused by streptozotocin, it leads to the formation of free radicals and superoxides with the help of xanthine oxidase in beta cells. These ROS kill beta cells and reduce insulin secretion, leading to hyperglycemia and Type II diabetes. The antioxidants present in moringa leaf bring down these reactive oxygen species, protect beta cells from being damaged, and keep hyperglycemia under control [25,127].

The effect of leaves extract in streptozotocin-induced diabeticrats has been investigated by Muzumbukilwa et al. [128], and they found that it can significantly improve the water intake, weight loss, fasting blood glucose (FBG), gamma-glutamylaminotrasminase and increase fasting plasma insulin (FPI) in diabetic rats. It also reduces the fasting plasma Alanine amino transaminase (ALAT), Aspartate amino transaminase (ASAT), and increases the plasma albumin in rats. The aqueous extract of moringa leaves also significantly reduced the blood glucose level in regular rats and maintained high blood sugar in the sub, moderate, and high diabetic rats [129].

Jimoh et al. [130] studied the antioxidant and inhibitory effects of *Moringa oleifera* and *Telfairia occidentalis* leaves against enzymes responsible for type II diabetes. In vitro α-amylase and α-glucosidase inhibitory assay were performed and found that moringa leaves showed significantly higher inhibitory effect for both assays (IC_50_ = 6.49 µg/mL (α-amylase) and IC_50_ = 4.73 µg/mL (α-glucosidase)) as compared to *Telfairia occidentalis* (IC_50_ = 10.60 µg/mL (α-amylase) and IC_50_ = 7.69 µg/mL (α-glucosidase). Additionally, moringa leaves also showed higher antioxidant activity. Higher phenolics in moringa leaves are responsible for their antioxidant activity and inhibition of both enzymes, showing their potential in managing Type II diabetes mellitus.

Li et al. [131] used RNA-seq and Methyl-seq technology to investigate the effect of methyl isothiocyanate on high glucose-induced diabetes nephropathy cell model in mouse kidney mesangial cells. Several epitomai and transcriptome alterations were revered by methyl isothiocyanate. RNA-seq data identified additional 20 canonical pathways with an inverse relationship between high glucose and methyl isothiocyanate. These pathways decrease the level of the negative effects of high glucose-induced kidney mesangial cells. A total of 173 and 149 DMRs were also identified between high glucose and low glucose groups, and high glucose and methyl isothiocyanate groups. The DMRs were reversed by methyl isothiocyanate. These alterations in pathways help to identify strategic therapeutic effects against high glucose.

The ethyl acetate fraction of moringa leaves at a 200 mg/kg body weight dose was orally administered to streptozotocin-induced diabetic rats for 30 days. Moringa leaves significantly increased body weight, food and water intake, blood glucose, insulin, and glycosylated hemoglobin. The hepatic marker enzymes (alanine transaminase (ALT), aspartate transaminase (AST), lactate dehydrogenase (LDH), alkaline phosphatase (ALP)), lipid profile level (triglycerides (TG), total cholesterol (TC), low density lipoprotein-cholesteriol (LDL-C)), pancreatic tumors necrosis factor-α (TNF-α) and interleukin-6 (IL-6), as well as serum interleukin-1β(IL-1β) levels were reduced in streptozotocin-induced diabetes rats fed with moringa leaves. Moreover, significant elevation in antioxidant enzymes (CAT, GST, SOD, GPx, GSH, Vitamin C and E) was observed. The increased antioxidant level and pro-inflammatory mediators’ inhibition proved moringa leaves as a potent anti-diabetic agent [132].

In another study, the antidiabetic effect of moringa leaves on the parotid gland of male albino rats was investigated. Moringa extract dose of 200 mg/kg body weight through the gastric tube was administered for three weeks. After moringa treatment, rats were euthanized with a heavy dose of halothane and various examinations of parotid glands were performed. The blood sugar level was significantly decreased. Light microscopy revealed that acinar cells, whose outlines with intracellular vacuolization and pyknotic nuclei were lost due to diabetes, started regaining their original shape and size. Similarly, fewer vacuoles and numerous parallel cisternae of the rough endoplasmic reticulum were seen in moringa-treated rats compared to multiple vacuoles and irregular rough endoplasmic reticulum arrangements by transmission electron microscopy. The comet assay showed a significant decrease in the tail moment of moringa-administered rats’ parotid glands, indicating lesser DNA damage [133].

Leone et al. [134] studied the α-amylase activity and postprandial glucose response in a Saharawi refugee camp by randomly choosing 17 people with diabetes and 10 healthy subjects and administering them with 20 g moringa leaves powder in the traditional diet. The α-amylase activity was decreased by 68.2 ± 3.2% compared to non-administrated subjects, with a minimum inhibition concentration of 120 ± 5 µg/mL. The postprandial glucose response peak increment was also lowered in diabetic patients after 90, 120, and 150 min of measurement. Additionally, the moringa administered patients showed a lower mean glycemic index (268 ± 18 mg/dL) than the control (296 ± 17 mg/dL). The high fiber content and secondary metabolites are responsible for the hypoglycemic index. High fiber content delayed glucose uptake in the intestine and gastric emptying time, whereas secondary metabolites are responsible for carbohydrate metabolism, thus inhibiting α-amylase and α-glucosidase enzymes.

### 4.5. Anti-Inflammatory Activity

Inflammatory diseases have long been the leading cause of morbidity and decreased labor worldwide. The usage of steroidal and non-steroidal medications for inflammatory disorders makes human organs highly prone to toxicity. *Moringa oleifera* is a herbal plant found to have anti-inflammatory activity in many studies. At a dose of 200 mg/kg, aqueous leaves extract exhibits an anti-inflammatory effect on animal models. The various active constituents present in the aqueous extract are responsible for inhibiting monocyte infiltration and fibroblast proliferation, which causes an anti-inflammatory effect. Pro-inflammatory cytokines produced by active monocytes trigger the TNF-α to enhance the inflammation of the cells by increasing endothelial cell adhesion of the neutrophils and lymphocytes [135]. Sharma and Singh [62] reported that 95% ethanolic leaf extract inhibits oedema development in carrageenan-induced paw oedema in albino mice at a dose of 1000 mg/kg body weight and reduces it by 79% after 5 h compared to the standard drug diclofenac sodium. Recently, diclofenac sodium and piroxicam showed a maximum reduction in egg albumin denaturation (IC_50_ value of 288.3, 253.8 µg/mL), whereas the methanolic and aqueous extract of moringa extract showed a maximum inhibition of proteinase activity (IC_50_ value of 199.3, 182.6 µg/mL) and ethyl acetate, and diclofenac sodium showed maximum stabilization of human red blood cells (IC_50_ value of 253.8 µg/mL) [70]. Aqueous extract of moringa leaves showed an almost similar anti-inflammatory response to ibuprofen (40 mg/kg) in rats administrated with egg albumin when paw circumference was measured after regular intervals. A medium dose of 424 mg/kg moringa leaves extract showed maximum inhibition by dwelling the inhibition of histamine, brady-kini or any mechanism due to phenolic compounds present in its leaves [136].

The impact of moringa leaves on inflammatory biomarkers in streptozotocin-induced male Wistar rats has been investigated by Oguntibeju et al. [75]. Rats have been split into four groups: non-diabetic, non-diabetic moringa-treated, diabetic, and diabetic moringa-treated. An amount of 55 mg/kg of streptozotocin was induced in rats, and they were treated with 250 mg/kg of methanolic extract of moringa leaves. The results showed that serum NF-kβ and IL-18 interleukin levels in the kidney and IL-1α, IL-18 in the liver were significantly reduced in moringa-treated diabetic rats. Additionally, Bcl-2 expression in both kidneys, as well as liver, was also upregulated. Enhanced cellular antioxidant potential due to moringa treatment helps minimize abnormal cell proliferation, and upregulation of inflammatory markers showed the anti-apoptotic and anti-inflammatory response of moringa leaves. Leutragoon et al. [137] also revealed that the ethyl acetate fraction of moringa leaves could regulate the NF-kβ pathways and suppress their nuclear translocation. mRNA expression of IF-1, IF-6, HelA, prostaglandin-endoperoxidase synthase-2 (PTGS2), and TNF-α was also suppressed. The expression of several other inflammatory mediators was also inhibited to show an anti-inflammatory response.

The aqueous moringa extract exhibited a substantial decrease in carrageenan and formaldehyde paw oedema and granuloma caused by a cotton pellet. Albino Wistar rats were given normal saline (5 mL/kg), dexamethasone drug (0.5 mg/kg), and aqueous moringa leaves extract (200 mg/kg). Moringa extract showed comparable results with 25.19% and 47.18% inhibition of carrageenan- and formaldehyde-induced paw oedema, respectively, and 41.48% inhibition of cotton pellet induced granuloma as compared to dexamethasone with 28.64%, 54.45%, and 58.71% inhibition. The presence of several active secondary metabolites and the release of mediators in all phases could be a possible reason for such anti-inflammatory action [135].

Suresh et al. [138] evaluated the anti-asthmatic effect of methanolic moringa leaf extract in ovalbumin-induced asthma in guinea pigs. 2.5 mg/kg of dexamethasone drug and 250 mg/kg and 500 mg/kg body weight of moringa leaves extract were given to ovalbumin-induced pigs from day 14 to 21 after their sensitization of day 14, and 0.5% ovalbumin was given from day 18 to 21 for 2 min. Drug and moringa leaves extract at both concentrations significantly reduced white blood cells, and the histamine level and tidal volume were maximized. Respiratory rate was also least affected, and histopathology studies showed the thinning of basement membrane airway smooth muscle which thickened due to ovalbumin exposure.

In another study, ethanolic moringa leaf extract showed a significant protective effect against diclofenac sodium-induced liver toxicity in male albino rats. Acute toxicity of leaf extract was measured with a dosing pattern from the dose of 500 to 4000 mg/kg body weight, and 300 mg/kg was found to be the maximum safest dose. Now, 150 and 300 mg/kg were the two doses given to diclofenac sodium-induced rats (100 mg/kg) through gastric gavages. Liver marker enzymes (alkaline transaminase, alkaline phosphatase, and aspartate transaminase) and bilirubin, creatinine, urea, and urease concentration were significantly decreased with moringa treatment. Antioxidant enzymes activity was increased, and nitric oxide activity was decreased. These advantageous effects of leaf extract might be attributed to the presence of bioactive compounds that aid in membrane stability and improved liver regeneration and reparative potential [139].

### 4.6. Cardiovascular Activity

Cardiovascular diseases involve several diseases in which heart and blood vessels related conditions are included. They were the leading cause of mortality worldwide. For hundreds of years, the treatment of cardiovascular disorders was conducted with medicinal herbs. This can be due to their ability to function as antioxidants, adrenoceptors, vasodilators, and antagonists of platelet-activating factors [7]. Moringa leaves showed remarkable effects on the circulatory system due to the presence of gossypetin, quercetagenin and proanthicyanadins and reduced mortalities due to various coronary heart diseases. Other phytochemicals present in leaves like niazinin and its derivatives and glucomoringinin were found to have a hypertensive bradycardiac effect [140].

Aekthammarat et al. [141] investigated the impact of aqueous extract of leaves on hypertensive rats. N-nitro-L-arginine-methyl ester was administrated to rats at a dose of 50 mg/kg/day for three weeks which increased their blood pressure and heart rate, and moringa extract was given at a dose of 30 and 60 mg/kg/day. The results indicated the dose-dependent decrease in blood pressure and tachycardia. The impairment of acetylcholine-induced relaxation and hyper-reactivity of adrenergic-mediated contraction was also reduced with treatment with moringa leaves extract. Moreover, it also showed antioxidant activity and other anti-hypertensive effects by inhibiting endothelium-dependent vasorelaxation.

Methanolic extract of moringa leaves also showed a protective role against oxidative stress in rats’ hearts under diabetic conditions. Moringa leaves extract was orally administrated to diabetic rats (streptozotocin-induced diabetes (30 mg/kg)) for 60 days at a dose of 300 mg/kg body weight. The blood glucose level, serum glucose, and glycated hemoglobin were significantly decreased, whereas plasma insulin was increased in moringa-treated rats. The level of antioxidant enzymes and glutathione content was increased in the rat heart. In addition, improved histopathology studies were also reported. GC-MS analysis also reported 12 different compounds in the extract, which might be responsible for reducing oxidative stress in the heart of diabetic rats [71].

Mabrouki et al. [142] reported that methanolic leaf extract also showed a cardiac ameliorative effect in high fat-induced obesity in male Wistar rats. Leaf extracts at a dose of 200 mg/kg and 400 mg/kg were orally administered to obese rats. The rats’ body weight and cardiac marker enzymes (cardiac catalase, glutathione peroxidase, and superoxide dismutase) were significantly reduced with leaf extract administration with a higher dose (400 mg/kg). Moreover, antioxidant enzyme activity was also improved. Histopathology studies revealed that necrosis areas were absent and myocardial fiber arrangement was the same as that of the control group.

A study was conducted on 66 participants (31 females and 35 males) to manage risk factors involved in cardiovascular diseases. All the participants received a supplement capsule consisting of *Moringa oleifera* (25 mg), *Bryophyllum pinnatum* (25 mg) and vitamin C (700 mg) for six months. After one month, the diastolic blood pressure of female participants was decreased by 3.26%. After three months, the average blood glucose level was reduced by 1.81%, but one male participant’s blood glucose improved significantly with a 61% reduction from baseline. LDL cholesterol level remained unchanged in male participants, and a 5.6% reduction was observed in females. On the other hand, HDL level was improved higher in male candidates from 1.03 to 1.24 mmol/L. *Bryophyllum pinnatum* and *Moringa oleifera* are responsible for hypotensive effects, and vitamin C is a good antioxidant. The hypotensive effects of moringa might be due to acetylated glycosides present in it [143].

Sieera-Campos et al. [144] evaluated the cardioprotective effect of moringa extracts on alloxan (120 mg/kg) induced diabetes in rats. The methanolic extract of moringa leaves was administered for 3 weeks at a 200 mg/kg dose. The results showed that glucose, triglycerides, AGEs, and glycated hemoglobin were significantly decreased from 415 ± 26 mg/dL to 125 ± 13 mg/dL, 198 ± 12 mg/dL to 145 ± 4 mg/dL, 6.8 ± 1.2 × 10^5^ AU/g protein to 1.7 ± 0.5 × 10^5^ AU/g protein and 10.4 ± 2.1% to 8.3 ± 3.7%, respectively, in moringa-treated diabetic rats as compared to diabetic rats. *Moringa oleifera* also inhibits the uncoupling of nitric oxide synthase (NOS) activity and upregulation of iNOS expression. The activity of paraoxonase was also induced by the binding of polyphenols present in the extract and decreased the affinity of other substrates. The ameliorative effect of aqueous leaf extract was compared with captopril (angiotension-converting enzyme inhibitor) and candesartan cilexetil (angiotensin receptor blocker) against Wistar rats exposed to petrol vapor. The rats were pretreated with 40 mg/kg aqueous leaf extract, 25 mg/kg captopril and 16 mg/kg candesartan cilexetil thirty minutes before exposure to petrol vapor. No significant differences were observed in all the three treated groups. The heart rate, hemolysis and percentage weight gain in treated rats were significantly decreased compared to petrol vapor-exposed rats. The possible mechanism of moringa leaves could be due to the protection of membrane integrity of erythrocytes which helps in the stabilization of cells and makes the cells osmotically resistant to redox effects of petrol [145].

### 4.7. Central Nervous System Activity

Many diseases are associated with the central nervous system, such as Parkinson’s, Alzheimer’s, Huntington’s, epilepsy and many more, which affect many different body activities such as inability to concentrate, movement, balance, memory loss, etc. Many medicinal plants have been reported for the treatment of these diseases related to the central nervous system. *Moringa oleifera* leaves were traditionally used to treat diseases such as epilepsy and Alzheimer’s of the central nervous system and cause anti-convulsant action because of the release of γ-amino butyric acid (GABA) [146].

It also showed significant enhancement in memory because of its action on neurons of the hippocampus. The sleeping time is also prolonged with moringa treatment as it increases the serum serotonin level, which activates the reticular activating system for better sleep [147]. Bhattachrya et al. [148] studied the dose-dependent effect of ethanolic extract moringa on locomotory activity and muscle relaxation by actophotometer and rotarod test, respectively. The six different groups of rats received normal saline (2 mg/kg) as a control group, Diazepam drug (10 mg/kg) and moringa extract (50, 100, 200 and 400 mg/kg) as an experimental group. A significant effect as central nervous system depressant and muscle relaxant was seen in the results. The presence of phytochemicals in the leaves readily crosses the blood–brain barrier and shows agonistic action on the GABA receptor complex, which might be responsible for this activity.

Al-Abri et al. [149] also studied moringa leaves extract’s motor and behavioral effects on mice. An amount of 0.9% saline solution was given to the control group orally and 100, 200, and 400 mg/kg of aqueous extract of moringa leaves to the experimental group for 14 days. The activity cage meter, hole board test and rota-rod treadmill were performed to study the motor and behavioral effects. Other thermal and chemical nociceptive tests performed were the hot plate, cold-water tail flick, writhing, and forced swimming. The significant dose-dependent anti-nociceptive activity was seen in both thermal and chemical tests. Mice with the highest dose (400 mg/kg) showed decreased exploration activity, neuromuscular coordination and mobility time in the forced swimming test, whereas no significant changes were seen in motor activity at different doses.

Mahaman et al. [150] evaluated the effect of moringa leaves on hyperhomocysteinemia (HHcy)-induced Alzheimer’s disease in rats. 400 µg/kg/day HHcy was induced through vena caudalis into rats for 14 days. The simultaneous and after injection treatment of methanolic extract of moringa was given to the rats as a preventative and curative treatment at a dose of 200 and 400 mg/kg/day. SCR1693 (1 mg/kg/day) was used a positive control. Moringa treatment decreased the neurodegeneration, and decreased synaptic proteins were also recovered. Tau phosphorylation and Aβ pathology induced by HHcy was decreased with moringa leaf extract treatment. Moringa leaves downregulate the calpain activity, which plays a crucial role in this process. Thus, the authors provide new insights into the treatment of Alzheimer’s disease, which is so far uncurable.

Oxidative stress-induced by hypoxia is the leading cause of brain dysfunction, and ethanolic extract of moringa leaves showed a significant effect on neurotoxic effects caused by hypoxia-induced by CoCl_2_. Five groups of fifty male rats (10 in each group) were designed: group 1 was the control, group 2 received an ethanolic extract of moringa (400 mg/kg) orally, group 3 received CoCl_2_ orally for 60 days at a concentration of 40 mg/kg, group 4 were administered with extract for 15 days prior and concurrently with CoCl_2,_ and group 5 were administered with extract for 15 days after CoCl_2_ treatment. GABA and monoamine neurotransmitter concentration was significantly reduced in hypoxia-induced rats. The expression of redox signaling genes was modified, and neuronal expression occupied by the glial fibrillary acidic protein (GFAP)-positive astroglia score was also elevated. The administration of moringa extract prior and concurrently with CoCl_2_ showed positive neurotoxic effects [151].

The neuroprotective effect of ethanolic extract of moringa leaves was also studied in quinine-treated rats’ myelin and neurofibers of the cerebellum. Seven different groups of rats were evaluated in which the first is the control group (group 1), groups 2–4 were administered with 10, 20 and 30 mg/kg quinine, respectively, group 5 was administered with 10 mg/kg quinine and 250 mg/kg leaf extract, group 6 was administered with 20 mg/kg quinine and 500 mg/kg leaf extract, and group 7 was administered with 30 mg/kg quinine and 750 mg/kg leaf extract. All the treatments were given for seven days. The results revealed that group 5 rats showed complete neural protection with neuronal regeneration and regular restoration of cerebellar cytoarchitecture, whereas the cerebellum of rats had minor structural damage in groups 6 and 7. Flavonoids in leaf extract were mainly responsible for this neuroprotective effect on the central nervous system [152].

## 5. Bioaccessibility and Bioavailability

Many studies have been performed on the nutritional and bioactive profiles of food matrices and by-products, but understanding their bioaccessibility and bioavailability is more important than knowing the quantity of specific compounds. The amount of polyphenols released for absorption from the food matrix in the digestive tract is known as bioaccessibility, whereas bioavailability provides information about the amount of digested compounds absorbed and metabolized by normal pathways [153]. In a study, the free phenolic compounds (gallic acid, caeffic acid, morin, kaemferol) and mono/oligosaccharides (mannose and stachyose) in the moringa leaves showed high bioaccessibility (6–210%). Gallic acid, chlorogenic acid, vanillin and rutin showed a higher bioaccessibilityat stomach level, whereas p-coumaric acid and quercetin showed higher value at the small intestine stage [154].

Dou et al. [155] reported that 2.48 (phenolics) and 2.20 (flavonoids) times were released during complete digestion. The oral digestion released a maximum amount of phenolics and flavonoids, i.e., 49.6% and 58.4%, respectively, whereas gastric digestion released a lower amount compared to oral digestion. The amount of phenolic acids was greater than flavonoids in the small intestine, which may be due to the easy degradation of flavonoids by digestive enzymes. The primaryphenolic compounds released during oral, gastric and intestinal digestion were 6,8-di-C-glucosylapigenin, catechin, ferulic acid and quercetin-3-O-β-D-glucoside, respectively.

Most of the flavonoids present in moringa leaves, i.e., quercetin, kaemferol, isorhamnetin and apigenin, exist in glycosylated form. Crespy et al. [156] reported that some flavonoids like quercetin were absorbed in the stomach of Wistar rats rather than their glycosidic forms. In another study, isoflavone aglycones were rapidly absorbed in the stomach rats and metabolites were found in blood plasma. The same was not observed in isoflavone glucosides when the stomach was solely a restricted absorption site [157]. Another effect of glycosylation was observed in rutin, which was absorbed later than quercetin in humans and rats [158,159]. The bioavailability of iron from moringa is extremely low because of high phytic acid [160]. The major folate forms found in moringa leaves are 5,6,7,8-tetrahydrofolic acid, 5-formyl-5,6,7,8-tetrahydrofolic acid, 5-methyl-5,6,7,8-tetrahydrofolic acid and 10-formylfolic acid [161]. The bioavailability of these folate forms in moringa leaves is high as compared to other leafy vegetables. *Moringa oleifera* folates showed 81.9% bioavailability in rat models when compared to synthetic folates [161]. However, due to diverse chemical structures, solubility, and interactions with the food matrix, the bioaccessibility and bioavailability of polyphenols and micronutrients in moringa leaves vary in different models. The susceptibility to digestion, methylation, fermentation and absorption in the gut may also vary. Hence, detailed in vitro and in vivo studies are required to investigate the bioavailability and metabolic pathways of moringa leaves’ polyphenols.

## 6. Dietary Application of Moringa Leaves in Food Products

Functional foods are becoming more important in today’s daily life because many chronic diseases have become prevalent, and these foods can eliminate or lessen their intensity. After several research studies regarding functional foods, it was concluded that these products find an important place among consumers’ requirements as they provide health benefitting properties [162]. Excessive free radical generation in the body may deteriorate large macromolecules, such as DNA, lipids and proteins, that cause numerous chronic diseases [163]. Antioxidants may thus be important in the prevention and treatment of chronic diseases. *Moringa oleifera* is gaining importance as an important functional food (Figure 2) due to the higher nutritional content of its edible portions and the presence of potent antioxidant compounds.

Moringa leaves, with high nutritional value, help combat malnutrition problems worldwide and may be used as nutraceuticals and functional foods due to natural antioxidants [9,29]. Recent studies showed that moringa leaves are widely used for the development of functional foods [164,165]. The nutrient content of several baked products was significantly increased with the addition of moringa leaves. Sengev et al. [166] fortified wheat flour bread with 5% leaves of moringa and found that there is a substantial increase in protein and crude fiber content of fortified bread, i.e., 54% and 56%, respectively, whereas other studies showed high crude fiber increase (88%) as compared to protein (17%) in fortified bread [167]. Tortilla chips fortified with 1, 3 and 5% moringa leaf flour showed higher protein content and a 50% increase in lipid content. Oleic and linoleic acids were the dominant fatty acids in fortified tortilla chips. TPC and antioxidant activity were significantly increased with the addition of moringa leaves [168]. Fombang and Saa [65] reported that functional tea formulated using moringa leaves showed a high amount of phenolic compounds and 81% inhibition in DPPH assay when samples contain a 1/20 mg/mL solid to liquid ratio at 97 °C and were processed for 35 min.

Moringa leaves also showed promising results on microbial food preservation and fermented food products. Tesfay et al. [169] investigated antifungal properties of moringa leaves and seed extracts against *L. theobromae*, *C. gloeosporiodes* and *A. alternata* strains when incorporated into an edible coating formulation for avocados. Ethanolic extract of leaf showed higher inhibition as compared to methanolic extract. Furthermore, fruits had a lower respiration rate and ethylene production. These findings are strongly linked with higher phenolic content in moringa leaves. *Mahewu*, supplemented with moringa leaves, showed a substantial increase in the beverage’s mineral, fat, and fiber content. Iron (350, 700 and 900%) and calcium (106, 214 and 287%) content were increased with 2, 4 and 6% moringa leaves, respectively. Beverages with 2% fortification showed the best sensory acceptability [167]. Many of the advantages of moringa leaves are ascribed to rich nutrients such as proteins and antioxidant compounds originating from vitamins and polyphenols, which make them important to a healthy and balanced diet and may be used as functional foods (Table 4).

## 7. Safety Aspects of Moringa Leaves

Generally, herbal preparations are considered safe and without adverse effects because they are considered natural products. Moringa leaves are highly recommended as natural dietary supplements because of their high nutritional value and low anti-nutritional factors. No adverse effects of moringa leaves have been observed in human studies so far. Moreover, many different formulations and preparations of leaves have been used worldwide as food, and no ill effects have been reported. The daily consumption of 70 g moringa leaf extract was considered safe with no toxicity [180]. In addition, several animal tests were examined for moringa leaves’ preparation toxicity. The toxicity of the aqueous extracts was evaluated in mice with oral administration of 6400 mg/kg and 1500 mg/kg intraperitoneally in the acute study, whereas 250, 500, and 1500 mg/kg were orally administered for 60 days in case of sub-chronic study. LD_50_ = 1585 mg/kg was the fatal dosage for mice. Histopathological and biochemical parameters showed no significant changes, and oral administration was regarded as safe consumption [181], whereas 400 to 2000 mg/kg body weight was confirmed as a safe dose in rats by Adedapo et al. [180]. The dose was given for 21 days, and blood cell count and serum enzyme level were evaluated as normal even at a higher dose (2000 mg/kg), and dose-dependent body weight decreased over the study.

Moodley [182] also reported that acute toxicity of moringa leaf powder at an oral dose of 2000 mg/kg to Sprague–Dawley rats was safe with no pathological symptoms and LD_50_ was found to be more than 2000 mg/kg. Similarly, in the sub-chronic study of Moodley [183], no change in clinical and net pathology was reported when leaf powder was orally given (90 days) at 1000 mg/kg per day dose. In another study, acute toxicity was evaluated in rats and rabbits with an infusion of 150 mg/mL of ethanol extract of moringa leaves by the intraperitoneal route until the death of the animal model occurred. The results showed that LD_50_ was rats and rabbits was 6616.67 mg/kg and 26,043.67 mg/kg, respectively [184].

In the case of humans, limited data have been published, and several trials mainly focused on hyperglycemia and dyslipidemia. Leone et al. [133] investigated the effect of dried moringa leaves powder on post-prandial blood glucose levels in refugees. A moringa leaves (20 g)-supplemented meal was given to 17 people with diabetes and ten healthy people. In moringa-treated diabetic individuals, the response to postprandial blood glucose peaked at 90, 120, 150 min with less increase than in control patients. No adverse effects on the subjects were evaluated, but the poor taste was the problem.

The safety and effects of moringa capsules in diabetic patients have also been investigated by Taweerutchana et al. [185]. Eight capsules of moringa leaf (4 g) or similar placebo capsules were given to subjects of an average age of 55 years (Haemoglobin A1C ≤ 9% and fasting plasma glucose ≤ 200 mg/dL) for four weeks before breakfast and dinner. No adverse effects of moringa leaves on the subjects were found. Furthermore, no significant effect of blood glucose was found in short-term studies. In another study, no side effects of leaves powder were found on postmenopausal women supplemented with 7 g of leaves powder per day for three months. However, antioxidant markers such as serum glutathione peroxidase, ascorbic acid, and superoxide dismutase increased significantly by 18%, 44.4%, and 10.4%, respectively, whereas malondialdehyde and superoxide dismutase fast blood glucose were decreased by 16.3% and 13.4%, respectively. Hemoglobin was also increased by 17.5% [186]. Studies have also revealed that moringa leaves absorb some heavy metals which might be toxic for human consumption; hence, care should be taken while these leaves are considered for medicinal as well as dietary purposes [187,188]. So, several human and animal studies concluded that various preparations of moringa leaves and aqueous extract were safe for consumption at specific doses and in the amount commonly utilized.

## 8. Conclusions and Future Aspects

*Moringa oleifera* leaves are recognized as important sources of micro-nutrients and phytochemicals that can be used for the development of nutraceuticals and functional foods. Moringa leaves contain key phytochemicals, which makes this plant an essential therapeutic agent with properties such as antioxidant, anticancerous, antimicrobial, antidiabetic, and anti-inflammatory properties. The dietary applications made this plant an important candidate for the development of major food products based on *Moringa oleifera* leaves, providing high nutritional value with acceptable sensory properties when used up to 10% in most food products. The food products based on these leaves showed more protein, dietary fibers, other nutrients, and important antioxidants. Moreover, consumption of moringa leaves within specific doses was also found to be safe. Overall, *Moringa oleifera* leaves are emerging as a prospective ingredient for developing food products that are nutritionally rich and therapeutically active. Furthermore, more clinical trials on the medicinal effects of moringa leaves are required to assess their safety for human consumption. Secondly, researchers need to extend their work on moringa polyphenols’ bioavailability and how complexing these polyphenols with other compounds affect their bioaccessibility.

## Figures and Tables

**Table 1:** Nutritional values of fresh and dried *Moringa oleifera* leaves as well as leaf powder.

Nutrients	Fresh Leaves	Dried Leaves	Leaf Powder
Calories (cal)	92	329	205
Crude protein (g)	6.7	29.4	27.1
Fat (g)	1.7	5.2	2.3
Carbohydrate (g)	12.5	41.2	38.2
Fiber (g)	0.9	12.5	19.2
Calcium (mg)	440	2185	2003
Potassium (mg)	259	1236	1324
Iron (mg)	0.85	25.6	28.2
Magnesium (mg)	42	448	368
Phosphorus (mg)	70	252	204
Copper (mg)	0.07	0.49	0.57
Sulphur (mg)	-	-	870
Vitamin A (mg)	1.28	3.63	16.3
Vitamin B_1_ (mg)	0.06	2.02	2.64
Vitamin B_2_ (mg)	0.05	21.3	20.5
Vitamin B_3_ (mg)	0.8	7.6	8.2
Vitamin C (mg)	220	15.8	17.3
Vitamin E (mg)	448	10.8	113
Chlorophyll (mg)	80	45	1268
Arginine (g/16 gN)	6%	1.78%	1.33%
Histidine (g/16 gN)	2.1%	0.716%	0.61%
Lysine (g/16 gN)	4.3%	1.637%	1.32%
Tryptophan (g/16 gN)	1.9%	0.486%	0.43%
Phenylalanine (g/16 gN)	6.4%	1.64%	1.39%
Methionine (g/16 gN)	2%	0.297%	0.35%
Threonine (g/16 gN)	4.9%	1.357%	1.19%
Leucine (g/16 gN)	9.3%	1.96%	1.95%
Isoleucine (g/16 gN)	6.3%	1.177%	0.83%
Valine (g/16 gN)	7.1%	1.413%	1.06%

Data adapted from [16,17] and all values are per 100 g of plant material.

**Table 2:** Polyphenolic compounds isolated from *Moringa oleifera* leaves.

Phenolic Class	Phenolic Sub-Class	Compounds	Extracting Solvent	References
**Phenolic acids**	Hydroxycinnamic acid derivatives	Chlorogenic acid	Methanol, 70% methanol	[32,33]
		Caffeic acid	Methanol, 70% methanol	[32,33]
		p-coumaric acid, p-coumaric acid ethyl ester	Methanol, 70% methanol	[32,33]
		Sinapic acid	Methanol;	[32]
		Ferulic acid, ferulic acid-4-O-glucoside	50% methanol	[32,34]
		1-Sinapoyl-2,2′-diferuloylgentiobiose	Methanol, 50% methanol	[34,35]
		Schottenol/Sitosterol ferulate	Methanol, 50% methanol	[34]
		24-methylcholestanol ferulate	Methanol, 50% methanol	[34]
		Feruloyl glucose	Methanol, 50% methanol	[34]
		2-S-glutathionyl caftaric acid	Methanol, 50% methanol	[34,35]
		1,2,2′-triferuloylgentiobiose	Methanol, 50% methanol	[34]
		Quinic acid, dicaffeoyl quinic acid, 3-O-caffeoylquinic acid, 4-O-caffeoylquinic acid, 3-caffeoylquinic acid 3-caffeoylquinic acid, 1,3-di-O-caffeoylquinic acid, 3,4-di-O-caffeoyquinic acid, 4,5-di-O-caffeoyquinic acid, coumaroylquinic acid isomer, 3-O-p-coumaroylquinic acid, feruloylquinic acid isomer, 3/4/5-sinapoylquinic acid, 3/4/5-feruloylquinic acid	Methanol, 50% methanol 70% methanol, 60% carbon dioxide expanded ethanol, Pressurized hot water, 80% ethanol	[33,35,36,37,38]
		1,2-diferuloylgentiobiose, 1,2-disinapoylgentiobiose	Methanol, 50% methanol	[35]
		24-methyllathosterol ferulate	Methanol, 50% methanol	[35]
		Verbascoside	Methanol, 50% methanol	[35]
		p-coumaroul glycolic acid	Methanol, 50% methanol	[35]
		Sitosterol ferulate	Methanol, 50% methanol	[35]
		Chicoric acid	Methanol, 50% methanol	[34]
		o-coumaric acid	70% methanol, Acetonitrile and 2N HCL	[33,39]
		trans-ferulic acid	70% methanol	[33]
		trans-cinnamic acid	70% methanol	[33]
		Salvianolic acid	Methanol, 70% methanol	[33]
		Caffeoyl shikimic acid	60% carbon dioxide expanded ethanol, Pressurized hot water	[36]
	Hydroxybenzoic acid derivatives	Protocatechuic acid	70% methanol	[33]
		Syringic acid	Methanol, 50% methanol, 70% methanol,	[33,34,35]
		Gallic acid, gallic acid ethyl ester, Gallic acid-4-O-glucoside	Methanol, 50% methanol, 70% methanol, 80% ethanol	[32,35,36,37,38]
		4-hydroxy-3-methoxybenzoic acid	Methanol, 50% methanol	[35]
		3-hydroxybenzoic acid	Methanol, 50% methanol	[34]
		Vanillin, vanillin glucoside	60% carbon dioxide expanded ethanol, Pressurized hot water	[32,36]
		Avenanthramide 2f		
	Hydroxyphenylacetic acid derivatives	3.4-dihydroxyphenylacetic acid	Methanol, 50% methanol	[35]
		Homoveratric acid	Methanol, 50% methanol	[35]
**Flavonoids**	Flavonol	dihydromyricetin-3-O-rhamnoside	Pressurized hot water; Acetonitrile and 2N HCL	[40]
		Quercetin, quercetin-3,7-diglucoside, quercetin-3-rhamanoside, quercetin-3-sophroside, quercetin-3-acetyl-glucoside, quercetin-3-glucoside, 3,7-dimethylquercetin, quercetin-3-O-rhamanoside, quercetin-3-O-galactoside, dihydroquercetin, dihydroquercetin-3-O-rhamnoside, quercitin-3-O-glucosyl-xyloside, quercitin-3-O-xylosyl-rutinoside, quercetin-malonylglucoside, quercetin-3-β-D-glucoside, quercetin-acetylglucoside, quercetin hydroxy-methylglutaronylglucoside	Methanol; Pressurized hot water; 70% methanol, 50%methanol 60% cabon dioxide expanded ethanol, Acetonitrile and 2N HCL, 80% ethanol	[32,33,34,35,36,37,38,39,40]
		Rutin	Methanol, 70% methanol	[32,33]
		kaempferol, kaempferol-3,7-diglucoside, kaempferol-3-glucoside, kaempferol-3-O-glucoside kaempferol-7-glucoside, kaempferol-3-O-rhamnoside, kaempferol-7-O-glucoside, kaempferol diacetyl-rhamnoside, kaempferol Acetyl-glucoside, kaempferol malonyl-glucoside	Methanol, Pressurized hot water; 70% methanol, 50% methanol; 60% cabon dioxide expanded ethanol, Acetonitrile and 2N HCL, 80% ethanol	[32,33,34,35,36,37,38,39,40]
		Morin	Methanol	[32]
		Procynadin dimer B7	Methanol, 50% methanol	[35]
		Methylgalangin	Methanol, 50% methanol	[35]
		Isorhamnetin-3-O-glucoside	60% carbon dioxide expanded ethanol, Pressurized hot water, 80% ethanol	[36,38]
		Silymarin	70% methanol	[33]
	Flavanols	Catechin, catechin-3-O-glucoside,	Methanol, 70% methanol; 50% methanol, Acetonitrile and 2N HCL	[32,33,35,39]
		Epicatechin	70% methanol	[33]
	Flavonones			
		Pinocembrin	Methanol, 50% methanol	[34,35]
		6-Geranylnaringenin	Methanol, 50% methanol	[34,35]
	Flavanone	Naringenin, 6-geranylnaringenin, naringenin-7-O-glucoside	Methanol, 50% methanol 70% methanol, Acetonitrile and 2N HCL	[33,34,35,39]
		Naringin, naringin-4-O-glucoside	Methanol, 50% methanol 70% methanol, Acetonitrile and 2N HCL	[33,34,35,39]
		Eriodictyol, eriodictyol-7-O-glucoside	Methanol, 50% methanol	[34,35]
		Eriocitrin	Methanol, 50% methanol	[34,35]
	Flavones	Hispidulin	Methanol, 50% methanol	[34,35]
		Apigenin, apigenin-8-C-glucoside, apigenin-7-C-glucoside, apigenin-6-C-glucoside, apigenin-7-O-glucoside	Methanol, 50% methanol 70% methanol, 80% ethanol	[33,35,37,38]
		Luteolin, luteolin-7-O-malonyl-glucoside, luteolin-7-O-glucoside	Methanol, 50% methanol 70% methanol	[34,35]
		Sinensetin	Methanol, 50% methanol	[34,35]
		Geraldone	Methanol, 50% methanol	[34,35]
		Tangeretin	Methanol, 50% methanol	[34,35]
		Isovitexin	70% methanol, 80% ethanol	[37,38]
		Acacetin	70% methanol	[33]
		Cirsiliol	70% methanol	[33]
		Cirsilineol	70% methanol	[33]
		Jaceosidin	Methanol, 50% methanol	[35]
		Myricitrin	Methanol, 50% methanol	[35]
	Dihydrochalcones	3-hydroxyphlorein-2-O-glucoside	Methanol, 50% methanol	[35]
		Phloretin-2-o-xylosyl-glucoside	Methanol, 50% methanol	[34]
	Isoflavonoids	6″-O-malonylgenistin	Methanol, 50% methanol	[34]
	Isoflavone	Genistin	Methanol, 50% methanol	[34,35]
		Biochanin A	Acetonitrile and 2N HCL	[39]
	Anthocyanins	Pelargonidin, pelargonidin-3,5-O-diglucoside, pelargonidin-3-O-glucosyl-rutinoside	Methanol, 50% methanol	[34,35]
		Pinotin A	Methanol, 50% methanol	[34,35]
		Delphinidin-3-O-sambubioside	Methanol, 50% methanol	[34,35]
		Delphinidin-3-O-glucoside	Methanol, 50% methanol	[34,35]
		Delphinidin-3-O-(6-acetyl-galactoside)	Methanol, 50% methanol	[34,35]
		Peonidin-3-O-(6-acetyl-galactoside)	Methanol, 50% methanol	[34,35]
		Cyanidin-3-O-xyloside	Methanol, 50% methanol	[34,35]
		Cyanidin-3-O-(6-malonyl-galactoside)	Methanol, 50% methanol	[34,35]
		Petunidin-3-O-(6-p-coumaroyl-glucoside)	Methanol, 50% methanol	[34,35]
		Malvidin-2-O-xylosyl-glucoside	Methanol, 50% methanol	[34,35]
**Thioglycosides (Glucosinolates)**		Glucomoringin isomer	60% carbon dioxide expanded ethanol, Pressurized hot water	[39]
**Other Polyphenols**	Lignans	Secoisolariciresinol-sesquilignan	Methanol, 50% methanol, ethyl acetate	[39]
		7-hydroxysecoisolariciresinol	Methanol, 50% methanol, ethyl acetate	[39]
		7-oxomatairesinol	Methanol, 50% methanol, ethyl acetate	[39]
		Isolariciresinol glucoside	Carbon dioxide expanded ethanol	[34,35]
	Alkylphenols	5-heptadecylresorcinol	Methanol, 50% methanol	[39]
		5-pentacosylresorcinol	Methanol, 50% methanol	[39]
		5-nonadecylresorcinol	Methanol, 50% methanol	[39]
		5-henicosylresorcinol	Methanol, 50% methanol	[39]
		5-pentacosenylresorcinol	Methanol, 50% methanol	[39]
		4-vinylphenol	Methanol, 50% methanol, ethyl acetate	[34,35]
	Hydroxycoumarins	Umbelliferone	Methanol, 50% methanol	[39]
		4-hydroxycoumarin	Methanol, 50% methanol	[34]
		Coumarin	Methanol, 50% methanol, ethyl acetate	[34]
		Mellein	Methanol, 50% methanol, ethyl acetate	[34]
	Hydroxyphenylpropenes	Estragole	Methanol, 50% methanol, ethyl acetate	[34,35]
		6-Gingerol	Methanol, 50% methanol	[39]
		Acetyl eugenol	Methanol, 50% methanol, ethyl acetate	[34]
	Tyrosols	Hydroxytyrosol, Hydroxytyrosol-4-O-glucoside	Methanol, 50% methanol, ethyl acetate	[34,35]
		3,4-DHPEA-AC	Methanol, 50% methanol, ethyl acetate	[39]
	Curcuminoids	Curcumin	Methanol, 50% methanol, ethyl acetate	[34]
		Demothoxycurcumin	Methanol, 50% methanol, ethyl acetate	[39]
	Furanocoumarins	Bergapten	Methanol, 50% methanol, ethyl acetate	[39]
	Hydroxycinnamaldehydes	Ferulaldehyde	Methanol, 50% methanol, ethyl acetate	[34]
	Naphtoquinones	1,4-naptoquinone	Methanol, 50% methanol, ethyl acetate	[39]
	Alkylmethoxyphenols	4-vinylsyringol	Methanol, 50% methanol	[39]
	Phenolic terpenes	Rosmanol	Methanol, 50% methanol	[39]
	Stilbenoids	Resveratrol, resveratrol-3-O-glucoside	Methanol, 50% methanol, Acetonitrile and 2N HCL	[34,35]

**Table 3:** Health benefits and mechanism of action demonstrated by *Moringa oleifera* polyphenols.

Health Benefits	Sample Type	Model Type	Result Summary/Mechanisms	References
**Antioxidant**	Subcritical ethanolic leaves extract of flavonoids	DPPH and FRAP assay	FRAP assay = 0.95–1.35 mmolFeSO_4_/mg DPPH assay (IC_50_ value) = 0.7440 mg/L	[57]
**Antimicrobial**	Aqueous leaf extract	Agar diffusion method	Inhibited the growth of *E.coli*, *S. typhi* and *P.aeruginosa* MIC: 10–20 mg/mL Large variation in antimicrobial activity (MIC for bacteria: 0.04–2.50 mg/mL and MIC for fungi: 0.16–>2.50 mg/mL) Coefficient of variability for bacteria in winter (75.2%) and summer (31.3%) Coefficient of variability for fungi in winter (19.2%) and summer (23.1%) Samples collected in winter had higher antifungal activity MBC: 20–40 mg/mL Inhibited the growth of some bacterial strains	[72]
	Acetone extract of 12 moringa tress harvested in different seasons	Two-fold serial dilution method		[73]
	Different extract of moringa leaves	Well diffusion assay		[35]
**Anticancerous**	Moringa leaves powder	Colorectal carcinogensis model (24 male mice)	Suppressed the AOM/DSS-induced colorectal carcinogenesis with 5% *w/v* of moringa dose. Ethanolic extract inhibits the proliferation of C4-II and HeLa cervical cancer cells due to decrease in NF-kB and Bcl-xL levels in these cells Moringa leaves synergize with vesicular stomatitis virus for cervical cancer treatments by altering the pathways involved in proliferation, apoptosis and antiviral responses. Moringa leaves increased BCL-2 expression in both liver and kidney tissues thus decreasing the expression of caspase 3, caspase 9 and NKFβ markers.	[32]
	Different extract of moringa leaves	Cervical cancer cell lines		[74]
	Methanolic extract	48 male wistar rats		[75]
**Antidiabetic**	Aqueous leaf extract	Albino rats	33.18% and 44.06% reduction in the blood sugar level of normoglycemic and hyperglycemic rats at a dose of 300 mg/kg after 6 h. Fasting plasma glucose (FPG) and post prandial blood glucose (PPPG) was reduced by 28% and 26%, respectively, with a daily dose of 8 g leaf powder for 40 days.	[76]
	Moringa leaves powder	Untreated Type-2 diabetic patients (30–60 years of age)		[77]
**Immunomodulatory activity**	Methanolic leaf extract	Wistar rats and swiss albino mice	Level of serum immunoglobulins increased, increase in adhesion of neutropenia, attenuation of cyclophosphamide-induced neutropenia. Cellular and humoral immune response stimulated at low doses. T helper cells, T cyctotoxic cells and B220^+^ cells were increased due to the presence of saponins and flavonoids. Restrict the development of herpes skin lesions and virus titers in brain were also reduced. Strong delayed type hypersensitivity (DTH) response to inactivated HSV-1 antigen. Elevated interferon-γ production by HSV-1 antigen. CD11b^+^ and CD49b^+^ subpopulations of splenocytes also enhanced.	[78]
	Aqueous leaf extract	Mus musculus mice		[79]
	Aqueous leaf extract	Ninety seven BALB/c female mice (Herpes simplex virus Type-I infected)		[80]
**Antiarthritic**	Ethanolic extract	Healthy Sprague–Dawley male rats (8–10 weeks old) with standard pellet diet	Moringa extract at a dose of 250 mg/Kg inhibits the CFA-induced arthritic paw edema. Significant decrease in arthritic index, the hematology profile was comparable to normal rats and significant higher effects than the CFA-control group.	[81]
**Antinociceptic effect**	Ethanolic extract	Healthy Sprague–Dawley male rats (8–10 weeks old) with standard pellet diet	Moringa extract at a dose of 500 mg/Kg showed a significant antinociceptic effect than indomethacin and the CFA-control group.	[81]
**Hypertension**	Moringa leaves powder	Sixty six male albino rats	Significant decrease in the systolic and diastolic blood pressure level of hypertensive rats, reduced the activity of arginase, acetylcholinesterase (AChE), phosphodiesterase-5 (PDE-5), angiotensin-1 converting enzyme (ACE) and higher antioxidant activity than hypertensive rats	[82]
**Anti-obesity effects**	Ethanolic extract	3T3-L1 *Mus musculus*, mouse cell lines	The expression of adipogenesis related genes were downregulated, decreased accumulated of triglyceride, induced apoptosis of adipocyte cells. Bax, a pro-apoptotic protein was upregulated, BCL-2 an antiapototic protein was downregulated, increased activity of caspase-3-activity.	[83]
**Anti-lipogenic effect**	Fermented *Moringa oleifera* leaves	Male peking ducks	Higher bodyweight, lower level of abdominal and subcutaneous fat, higher serum insulin. Hepatic lipid, triglycerides, low density lipoprotein cholesterol decreased, whereas high density lipoprotein cholesterol and leptin increased. Expression of lipogenesis-related genes in abdominal fat were downregulated.	[84]

DPPH: 2,2-dipheyl-1-picrylhydrazyl, FRAP: Ferric reducing antioxidant power, MIC: Minimum inhibitory concentration, MBC: Minimum bactericidal concentration, AOM/DSS: Azoxymethane/Dextran sodium sulfate, NF-kB: Nuclear factor kappa-B, Bcl-xL: B-cell lymphoma extra large, BCL-2: B-cell lymphoma 2, HSV-1: Herpes simplex virus-1, CFA: complete freund’s adjuvant.

**Table 4:** Applications of *Moringa oleifera* leaves as a functional ingredient in food products.

Food Stuff	Concentration of Leaves Used (%)	Functional Advantage	Related Bioactive Compounds	Reference
**Snacks**	1	High in mineral content and protein, less fat	-	[170]
**Vegetable soup powder**	8.5	Longer shelf life Enhanced nutritional quality	Protein, fiber, vitamin D and C and minerals	[171]
**Cattle feed**	25	Higher milk yield, milk fat, lactose content	-	[172]
**Yoghurt**	0.5–2	Higher nutritional value	-	[173]
**Bread**	5	Better nutritional quality with less organoleptic change	Protein, fiber and minerals	[166]
**Cookies**	10–20	Higher protein content with acceptable sensory qualities	-	[174]
**Yoghurt**	0.5	Acceptable Sensory qualities	-	[175]
**Sour cream**	600, 800 and 1000 ppm	Higher protein, acidity and peroxide value Acceptable sensory quality during storage	-	[176]
**Ready to eat snacks**	20	Decrease in antinutritional factors	Phenolic compounds, saponins and phytic acid	[177]
**Amala**	2.5–10	Protein content increase by 48% at 10% leaf powder concentration mineral content also increased but addition above 2.5% adversely affect sensory attributes	-	[178]
**Cattle feed**	10.85	Higher nutritional value, a higher concentration of total ruminal volatile acids, greater relative expression of microbial genes.	-	[179]

## Data Availability

Data sharing not applicable.
